# A pilot clinical validation study of a self-collected vaginal swab device for the detection of *chlamydia trachomatis* in women

**DOI:** 10.3389/fbioe.2022.1008761

**Published:** 2022-10-04

**Authors:** Michael Muljadi, Chao-Min Cheng, Chung-Yao Yang, Ting-Chang Chang, Ching-Ju Shen

**Affiliations:** ^1^ Institute of Biomedical Engineering, National Tsing Hua University, Hsinchu, Taiwan; ^2^ Hygeia Touch Inc, Taipei, Taiwan; ^3^ Division of Gynecologic Oncology, Department of Obstetrics and Gynecology, Chang Gung Memorial Hospital at Linkou, College of Medicine, Chang Gung University, Taoyuan, Taiwan; ^4^ Department of Obstetrics and Gynecology, Kaohsiung Medical University Hospital, Kaohsiung Medical University, Kaohsiung, Taiwan

**Keywords:** *chlamydia*, std, naat, PCR, vaginal swab, self-collection tool, gynecology

## Abstract

*Chlamydia trachomatis* (*C. trachomatis*) is one of the most prevalent preventable sexually transmitted diseases (STDs) in the world. In women, *C. trachomatis* infection can lead to long-term complications such as pelvic inflammatory disease (PID), and other related conditions such as ectopic pregnancies and even tubal factor infertility. These complications are preventable given early detection and clinical intervention, but these efforts are often hampered by asymptomatic silent infections, and non-compliance to screenings for STDs. Some women do not get tested out of concerns for violation of privacy, and fear of discomfort. Clinicians often use a multitude of tests to determine if a patient is infected by *C. trachomatis*, including a Polymerase Chain Reaction (PCR) test of First catch urine (FCU) samples. However, these tend to be inconvenient to store and transport, as they carry risk of spillage and have stringent refrigeration requirements. Moreover, given the gold-standard recommendations set forth by the Centres for Disease Control (CDC), the current technique can be inconvenient in remote areas where refrigeration and transport may not always be reliable. The current study therefore looks at the potential of a self-collected vaginal swab device that relies on Nucleic Acid Amplification Tests (NAATs), is dry-stored, and does not require refrigeration, to detect the presence of *C. trachomatis* in women. The study found evidence to suggest that the self-collection device has the potential to aid clinicians in the diagnosis of *C. trachomatis* in women when compared to doctor-collected vaginal discharge samples as the designated standard, FCU, and blood serology. Moreover, as a self-collection device it has the potential to break down some of the barriers to STD screening especially in young women, such as violation of privacy. The device therefore has a potential to encourage screening and therefore a potentially effective tool in the fight against the spread of preventable sexually transmitted diseases.

## Introduction

Sexually Transmitted Diseases (STDs) continue to be a worldwide public health concern over the last couple of decades. Back in the year 2011, the bacterial STD *Chlamydia Trachomatis* (*C. Trachomatis*) made up the largest number of cases ever reported to the Centre for Disease Control (CDC) for any condition at 1.4 million infections worldwide (Adams et al., 2013). Also, another bacterial STD, *Neisseria Gonorrhoea* (*N. Gonorrhoea*) averaged 104 cases per 100,000 population, the highest rates of which were in adolescents aged 15–24 years old. Fast forward to the year 2019, the World Health Organisation reported an estimate of more than 376 million cases of four curable bacterial sexually transmitted infections: *chlamydia*, *gonorrhoea*, *trichomoniasis*, and *syphilis* (Rowley et al., 2019). Despite the availability of early treatment strategies, bacterial sexually transmitted infections remain endemic worldwide.

Apart from safe-sex practices, routine screening is known to be an effective strategy in preventing complications related to STDs, as well as ensuring early interventions where necessary. Both *C. Trachomatis* and *N. Gonorrhoea* in particular, have been associated with female infertility including tubal factor infertility, and pelvic inflammatory disease (PID) (Tsevat et al., 2017). This is in contrast to two other organisms: *Mycoplasma Genitalium* (*M. Genitalium*) and *Trichomonas Vaginalis* (*T. Vaginalis*), which had limited evidence associating them with the pathology of infertility. Despite benefits associated with it, adolescents are known to be less than compliant when it comes to screening. This was also illustrated in a study by Rietmeijer and others, where only less than half of at-risk youth was found to seek preventative STD screening services (Rietmeijer et al., 1998). This less than satisfactory compliance to screening can be attributed to fear and lack of knowledge. Adolescents are known to avoid pelvic examinations due to fears of distressing results, physical discomfort, and invasion of privacy (Millstein et al., 1984). The absence of symptoms and low self-assessed risk have also been found to be reasons given by young women for not getting tested (Langille et al., 2008), despite *chlamydia*’s tendency to be asymptomatic in most women–up to 70%–75% (Papp et al., 2014; Patel et al., 2018). These have prompted studies that look at better ways to improve screening compliance among young women to prevent infertility and facilitate early intervention.

Nucleic Acid Amplification Tests (NAATs) detect the presence of a particular organism through a detection of specific nucleic acid sequences in a sample. It has been shown to be superior compared to traditional methods such as culture plates, which requires prolonged periods of incubation and a significant amount of resources to execute (Schachter, 1986). Cultures rely on the presence of viable pathogens, requiring refrigeration and possibly a liquid buffer, complicating the transport of samples from clinic to lab. NAATs are currently the recommended gold standard (Papp et al., 2014) for both *chlamydia* and gonorrhoea because of its comparable specificity to culture. In addition, because they do not rely on viable pathogens to perform, NAATs allow for the self-collection of samples and potentially removing existing barriers to STD screening. In *chlamydia* asymptomatic women for example, detection *via* NAAT has been shown to be most accurate through self-collected vaginal swabs when compared to cervical and urine samples (Wiesenfeld et al., 1996; Schachter et al., 2003). Schachter also reported no observable difference in results between self-collected and clinician-collected vaginal swabs. Shafer and others also reported similar results, with self-collected vaginal swabs identifying the highest number of positive results among single specimens with the inclusion of *N. Gonorrhoea* cases, where endocervical and urine specimens performed particularly poorly (Shafer et al., 2003). There is also evidence to suggest that NAATs remain accurate even through non-traditional screening programs outside of clinics and medical centres. Masek and others compared the performance of three NAATs using self-collected vaginal swabs in an internet-based screening program and found that *C. trachomatis* and *N. gonorrhoea* had Polymerase Chain Reaction (PCR) sensitivities of 100%, while specificities were found to be 99.3% and 98.8% respectively (Masek et al., 2009).

Apart from accuracy, there is also evidence to suggest that self-collected vaginal swabs are generally well accepted among young women. Doshi and others reported a 90.4% collection rate of self-collected vaginal swabs, coupled with documented acceptability of the swabs for *chlamydia* screening (Doshi et al., 2008). 99% of participants in Wiesenfeld’s study also reported that self-collected vaginal swabs were easy to perform, and 84% of participants reported preference for it over gynaecological examinations (Wiesenfeld et al., 2001). Even more than acceptance, there is also evidence to suggest that self-collected vaginal swabs may improve STD screening compliance. 94% of Wiesenfeld’s study participants stated their willingness to undergo screening at more frequent intervals given that self-collection was made available. Even when done through a non-conventional home sampling strategy, they have also been associated with a reduction in *C. trachomatis* prevalence and lower proportion of reported cases of PID (Østergaard et al., 2000).

However, most self-collected vaginal swabs rely on immediate refrigeration on site (Doshi et al., 2008) or storage into a buffer or NAAT transport fluid by the participants themselves (Wiesenfeld et al., 1996; Wiesenfeld et al., 2001; Shafer et al., 2003; Masek et al., 2009). This complicates the collection procedure for both patients and medical personnel due to risk of spillage, refrigeration requirements, and transport from clinic or home to the lab. According to the “*Recommendations for the Laboratory-Based Detection of Chlamydia trachomatis and Neisseria gonorrhoeae–2014*” by the Centres for Disease Control and Prevention, the use of FDA-cleared NAATs such as the Abbot RealTime CT/NG requires stringent specimen transport and storage conditions; specimens from asymptomatic women needs to be stored between 2 and 30°C and used within 14 days, while specimens from symptomatic women has to be thaw frozen and stored at the same temperature range (Papp et al., 2014). Whilst this may still be possible in urban and high-income populations, it presents a challenge in remote areas where refrigeration may not always be available, especially during specimen transport. This can be crucial considering that many *C. trachomatis* infections also occur in countries where public infrastructures may be limited (O’Connell and Ferone, 2016). The recommendation has also noted that whilst first catch urine specimens are acceptable, it might detect up to 10% fewer infections compared to vaginal swab samples (Papp et al., 2014). Moreover, there are no commercially available NAAT vaginal swab test kits approved by the Food and Drug Administration (FDA). Therefore, there is opportunity in this space to engineer a device for the self-collection of vaginal swabs for use in the screening of *C. trachomatis* that incorporate the advantages of NAATs, with the addition of ease of storage and transport for patients through the absence of transport buffer or fluid, and refrigeration requirements. This user-friendly addition to self-collected vaginal swabs can potentially further reduce barriers to STD screening by providing a comfortable, painless, private, and convenient alternative to conventional screening procedures such as clinician-collected swabs, and more invasive ones such as pelvic and speculum examinations.

The current study aims to look at the potential clinical feasibility of a self-collected vaginal swab device that utilises PCR NAAT technology and the advantages of dry storage in the absence of a buffer for the qualitative detection of *C. trachomatis*. Results were tested against *C. trachomatis* PCR of doctor-collected vaginal swab samples for validity. Additionally, results were compared with currently utilised diagnostic tests used to aid clinicians at the Kaohsiung Medical University Hospital (KMUH) obstetrics and gynaecology outpatient clinic in making *C. trachomatis* diagnosis. These include IgM and IgG *C. trachomatis* antibodies from blood samples, and *C. trachomatis* PCR from First catch urine (FCU) samples. *C. trachomatis* serology is known to play a role in the investigation of infection incidence, with the potential to be a biomarker for scarring sequelae such as PID (Woodhall et al., 2018). They have also been suggested for use as a potential marker for active *C. trachomatis* infection (Perhar et al., 2020). On the other hand, FCU samples are included for comparison because it is one of the recommended specimens to be used with NAAT’s and not as invasive (Papp et al., 2014). The comparisons done against the self-collected vaginal swab were also made in terms of accuracy, and ease of transport and use.

## Materials and methods

### Patients

A total of 31 female patients aged between 20 and 65 years old attending Kaohsiung Medical University Hospital’s (KMUH) obstetrics and gynaecology outpatient clinic in southern Taiwan were included in the study following formal consent. Project approval was obtained from the Institutional Review Board (IRB, #KMUHIRB-F (I)-20220090). Limited demographic information such as date of birth and symptoms were also obtained from patients in conjunction with formal examination by a clinician. Inclusion criteria was set for cases of outpatient consultations due to vaginitis or infertility and which are suspect for *chlamydia* and gonorrhoea. Clinically diagnosed non-bacterial vaginitis and pregnancy cases were excluded from the study. Clinically assessed symptoms were also subsequently classified using the World Health Organisation’s (WHO) International Classification of Diseases (ICD) (Organization, 1978).

### Self-collection tool

**FIGURE 1:**
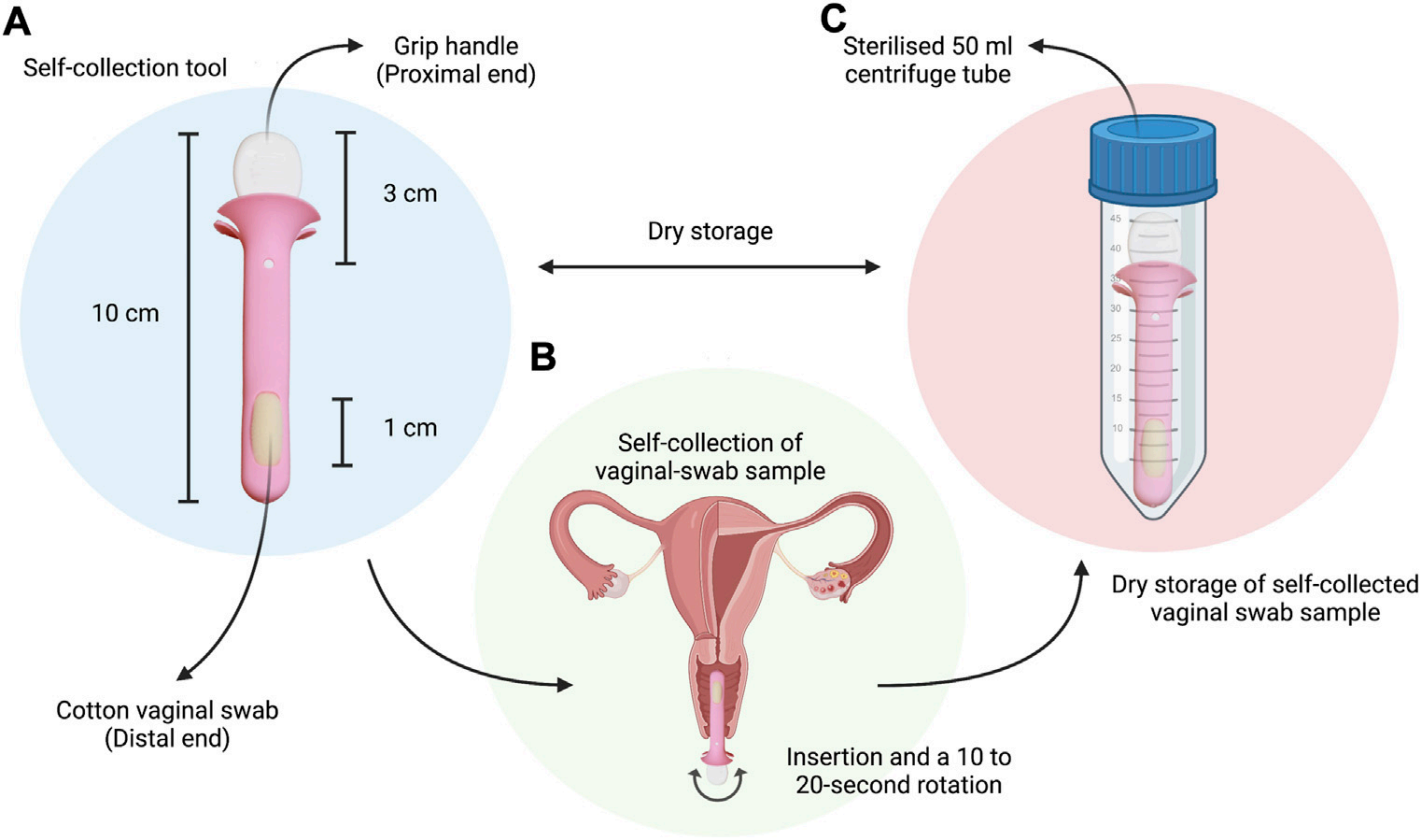
Simple schematic highlighting: **(A)** The vaginal swab self-collection tool given to study participants, with a total designated length of approximately 10 cm, a 3 cm handle on the proximal end for comfortable grip, and a 1-cm cotton vaginal swab embedded on the distal end; **(B)** The self-collection procedure instructed to study participants during visit to the outpatient clinic (insertion and; **(C)** The dry storage of samples in a sterilised 50 ml centrifuge tube for transport to the lab for further processing and analysis. Created with BioRender.com (https://app.biorender.com/).

The tool is an approximately 10 cm length rod with a diameter of approximately 1 cm. It has a small grip handle of approximately 3 cm in length from its root embedded on the proximal end, and a cotton swab of approximately 1 cm length on the distal end for the collection of vaginal discharge samples (Figure 1).

### Sample collection and storage

First-catch-urine samples, blood samples, and clinician-collected vaginal swabs were collected from the patients. Clinician-collected vaginal swab samples were obtained during pelvic examination using a wet, sterile cotton swab of approximately 10 cm in length. The cotton swabs were then stored in a sterile specimen jar for storage and transport. Subsequently, patients were also given a tool for the self-collection of vaginal swabs. Patients were instructed to insert the swab at least approximately 2.5 cm into the vagina, followed by a 10–20 s rotation, and finally storage of sample in a sterile 50 ml centrifuge tube for transport. These instructions were adapted from that of the commercially available Abbott RealTime CT/NG testing kit for *Chlamydia trachomatis* from Abbott Molecular Inc.

### Sample processing

**FIGURE 2:**
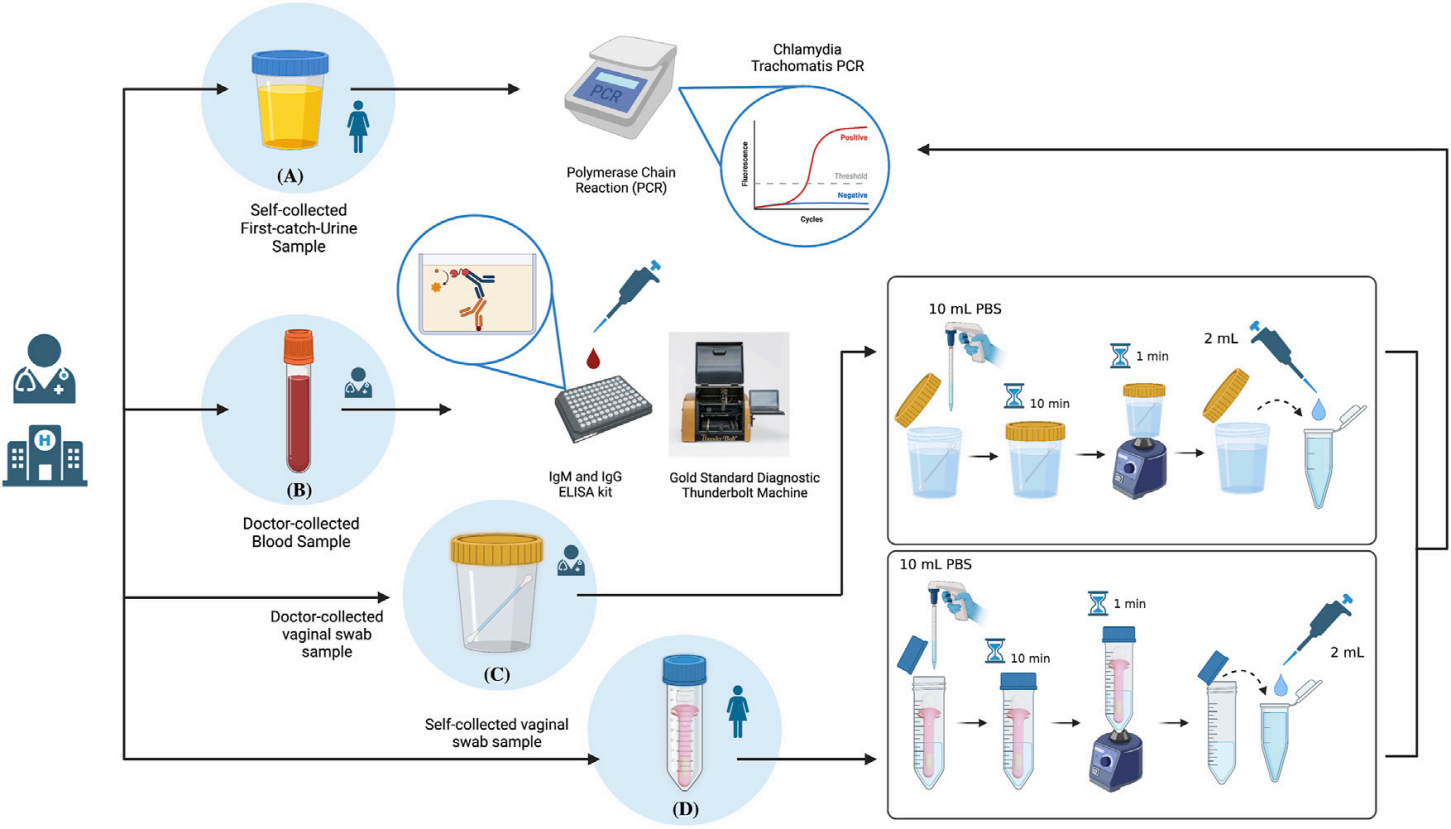
Schematic summary of the sample processing procedures highlighting: **(A)** NAAT Polymerase Chain Reaction (PCR) analysis of self-collected First catch-urine (FCU) samples; **(B)** IgM and IgG antibody analyses of doctor-collected blood samples *via* ELISA; **(C)** sample preparation for NAAT PCR analysis of doctor-collected vaginal swab samples; **(D)** sample preparation for NAAT PCR analysis of dry-stored vaginal swab samples collected by the self-collection tool. Created with BioRender.com (https://app.biorender.com/).

Both self-collected and doctor-collected vaginal swab samples were transported to the lab followed by processing (Figure 2). Special refrigeration was not arranged during specimen transport, as Nucleic Acid Amplification Tests (NAATs) do not depend on viable pathogens, unlike analyses using traditional plate culture methods. This is also because the current device was tested for its ability to detect *C. trachomatis* in the absence of refrigeration as a comparative advantage against the gold standard specimen transport procedures (Papp et al., 2014). First-catch-urine samples were also transported to the lab for *C. trachomatis* NAAT. IgG and IgM antibody tests of the blood samples were conducted at the Kaohsiung Medical University Hospital’s in-house laboratory following collection *via* the *C. trachomatis* IgG and IgM ELISA kits, analysed using the ThunderBOLT^®^ automated ELISA system by Eurofins Technologies.

Both self-collected and doctor-collected vaginal swab samples were first identically introduced to 10 ml of sterilized Phospho-buffered Saline (PBS) in its original specimen tube; a 50 ml centrifuge tube for self-collected vaginal swab samples, and standard clinical use specimen jar for clinician-collected vaginal swab samples. Samples were let incubate in PBS for 10 min, followed by 1 min of mixing using the vortex machine. 2 ml of both samples were collected for *Chlamydia trachomatis* qualitative processing using an *in vitro* Polymerase Chain Reaction (PCR) assay (Abbott REALTIME CT/NG). Remaining samples were stored for future use under −20°C.

### Data analysis

*C. trachomatis* PCR results from doctor-collected vaginal swab samples was considered the designated gold standard to determine positive vs negative cases. This is consistent with recommendations set forth in the “*Recommendations for the Laboratory-Based Detection of Chlamydia trachomatis and Neisseria gonorrhoeae–2014*” by the Centres for Disease Control and Prevention (Papp et al., 2014). Data were exported to both Microsoft Excel and IBM SPSS Statistics ver. 26. Fisher’s Exact Test was used to test the significance of association between the designated gold standard (doctor-collected *C. trachomatis* vaginal swab samples) and the four test variables (self-collected vaginal swab, FCU PCR, blood IgM and IgG), as it has been shown to be appropriate at lower sample sizes (Connelly, 2016). Results are considered statistically significant when statistical tests yield a *p*-value of *p* < 0.05.

## Results

### Patient demographics and frequencies

Doctor-collected and self-collected vaginal swab samples were collected from all 31 (*n* = 31) of the recruited study participants. FCU sample and blood sample was not collected from one of the study participants, making the total of FCU and blood samples at 30 (96.77%) valid cases. Age data was also not collected from two participants. Sample mean age of participants (*n* = 29) was found to be 32.31 years, and at a median of 31 years. Sample standard deviation was 7.56 years, and the minimum and maximum age of study participants were 21 years and 51 years respectively.

Out of the 31 valid cases for both doctor-collected and self-collected vaginal swab samples, 87.1% (27 cases) of cases were *C. trachomatis* negative while 12.9% (4 cases) were positive. Additionally, there were 30 valid cases for FCU, blood IgM, and blood IgG. 90% (27 cases) of the total FCU *C. trachomatis* PCR results were negative, while 10% (3 cases) were positive. 96.7% (29 cases) of the total *C. trachomatis* blood IgM results were negative, while 3.3% (1 case) were positive. Finally, according to blood IgG results, 60% (18 cases) were negative while 40% (12 cases) were positive.

### Cross-tabulation of results

**FIGURE 3:**
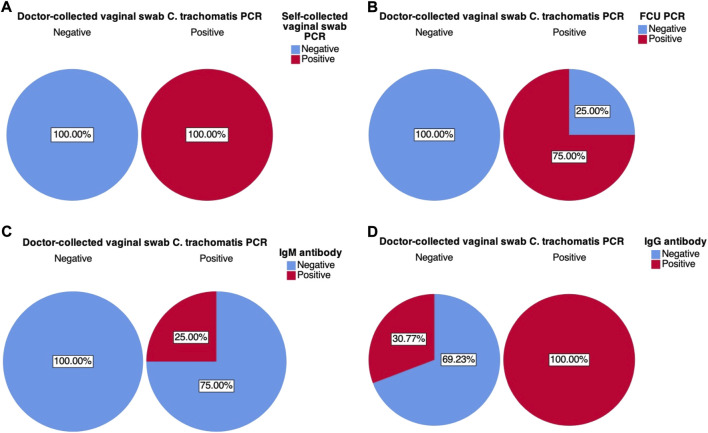
Graphical pie-chart visualisation of cross-tabulations between doctor-collected vaginal swab *C. trachomatis* PCR results and: **(A)** Self-collected vaginal swab *C. trachomatis* PCR; **(B)** First catch-urine (FCU) *C. trachomatis* PCR; **(C)** Blood *C. trachomatis* IgM antibody ELISA; **(D)** Blood *C. trachomatis* IgG antibody ELISA. Percentages were calculated based on counts within doctor-collected sample PCR results.

**FIGURE 4:**
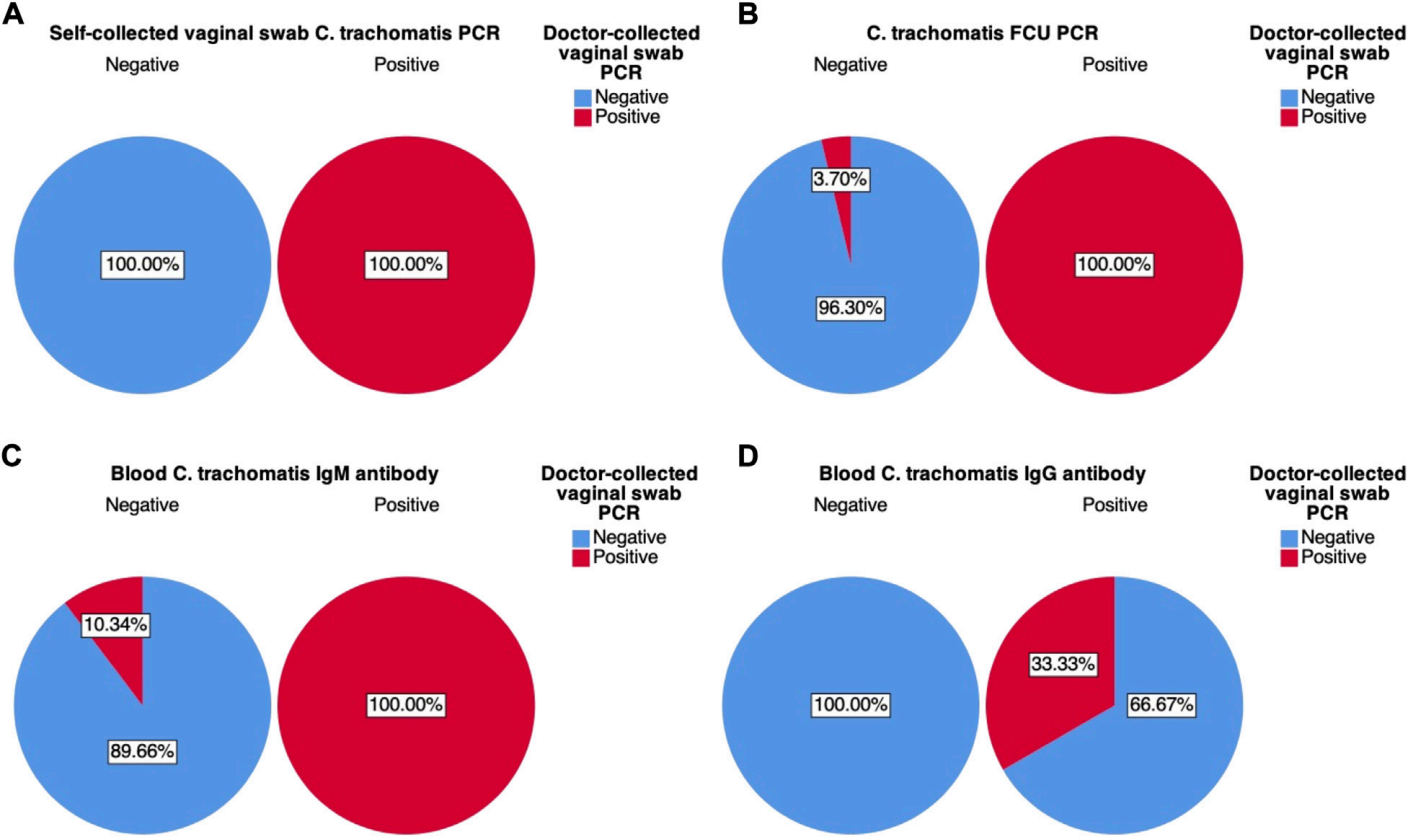
Graphical pie-chart visualization of cross-tabulations between doctor-collected vaginal swab *C. trachomatis* PCR results and: **(A)** Self-collected vaginal swab *C. trachomatis* PCR; **(B)** First catch-urine (FCU) *C. trachomatis* PCR; **(C)** Blood *C. trachomatis* IgM antibody ELISA; **(D)** Blood *C. trachomatis* IgG antibody ELISA. Percentages were calculated based on counts within each of the four test variables.

Cross-tabulation of each of the four test variables: *C. trachomatis* FCU and self-collected vaginal swab sample PCR, and blood IgM and IgG antibody, against the designated gold standard doctor-collected PCR revealed that self-collected vaginal swabs was found to have the highest sensitivity (100%) and specificity (100%) (Figures 3, 4). First-catch urine (FCU) PCR was found to have a sensitivity of 75% (false negative rate–type 2 error rate–of 25%), and a specificity of 100%. Blood IgM antibody was found to have a sensitivity of 25% (false negative rate–type 2 error rate–of 75%) and a specificity of 100%, while blood IgG antibody was found to have a sensitivity of 100% and a specificity of 69.23% (false positive rate–type 1 error rate–of 30.77%).

Further analysis of variation within each of the four test variables also revealed their positive (PPV) and negative predictive values (NPV), therefore also their false discovery (FDR) and false omission rates (FOR). Self-collected vaginal swab sample PCR was found to have a PPV and NPV of 100%. FCU PCR was found to have a PPV of 100%, while NPV was found to be 96.37% (FOR of 3.70%). Blood IgM antibody was found to have a PPV of 100% and an NPV of 89.66% (FOR of 10.34%), while blood IgG antibody was found to have a PPV of 33.33% (FDR of 66.67%) and an NPV of 100%.

### Statistical analysis

Cross-tabulations of each of the four test variables against *C. trachomatis* PCR results from doctor-collected vaginal swab samples were also statistically tested using the Fisher’s Exact Test, as one or more cells in the 2 × 2 contingency tables had counts of less than 5. The test revealed that there was sufficient evidence to suggest a statistically significant association between the *C. trachomatis* PCR of doctor-collected vaginal swab samples and the self-collected samples (*p* < 0.001). This was also evident when associated with *C. trachomatis* FCU PCR (*p* = 0.001), and blood IgG antibody (*p* = 0.018) at a critical *p*-value of *p* < 0.05. However, there was insufficient evidence to suggest significant association with blood IgM antibody (*p* = 0.133) at the same critical *p*-value.

## Discussion

Based on the available statistical evidence, and sensitivity and specificity analysis, there was evidence to suggest that there is potential for the vaginal swab self-collection device to become an alternative diagnostic tool to aid clinicians in the screening of *chlamydia trachomatis*. The performance of the current device was studied in comparison with a couple of methodologies currently used by outpatient clinicians at the Kaohsiung Medical University Hospital department of obstetrics and gynaecology in making a diagnosis for *C. trachomatis*. These included antibody IgM and IgG analysis of blood samples, and NAAT PCR analysis of FCU samples. As a designated gold standard, NAAT PCR results of doctor-collected vaginal swab samples were used as a comparison point in both statistical analysis, and sensitivity and specificity analysis. Based on results from Fisher’s exact test of association, there was sufficient statistical evidence to suggest an association between doctor-collected swab NAAT PCR results and self-collected swab PCR, FCU PCR, and blood IgG antibody. There was insufficient statistical evidence to suggest an association between doctor-collected swab PCR and blood IgM antibody, suggesting independence between the two samples.

These associations were further explored through contingency tables of these variables, the visualisations of which are presented in Figure 3 and Figure 4. Based on Figure 3’s charts A to D, self-collected vaginal swab samples showed the greatest number of sensitivity and specificity at 100%. Both FCU PCR and IgM also showed superior specificity (100%, Figures 3.B,C), indicating that there is a high probability of obtaining a negative result on the condition that results were truly negative–in this case represented by the designated gold standard doctor-collected vaginal swab PCR. Sensitivity results appeared less promising for these test variables; with FCU PCR having a higher probability (75%, Figure 3B) of a positive compared to IgM antibody (25%, Figure 3C) when results were truly positive. This suggests that while both these tests may be reliable in reflecting true negative results, they might not be as sensitive to true positives, potentially giving false negative results. Moreover, Fisher’s exact test failed to suggest association between results from blood IgM and doctor-collected vaginal swab PCR, suggesting that these associations might be due to random chance. These results further discount the test’s utility in *C. trachomatis* diagnosis. The potential implications of relying on these results is that they may underreport true cases of *C. trachomatis*, potentially leading to misdiagnosis and untreated cases, and therefore its continued spread. This can also potentially explain the disease’s worldwide endemic status as reported by the WHO, as clinicians continue to use some of these tests to aid in their diagnosis. Blood IgG results were the opposite, where there is a high probability of obtaining a positive result given that results are truly positive (100%, Figure 3D), while there is a 30.77% probability of obtaining false positives when results are truly negative (69.23% specificity). The implications of a false positive result may be equally if not more undesirable than false negatives, as this means that women may be falsely treated with antibiotics despite not carrying the disease.

Positive (PPV) and negative (NPV) predictive values of the test variables were also visualised in charts A–D of Figure 4. Similar to sensitivity and specificity results, both PPV and NPV were strongest for self-collected vaginal swab samples (100%, Figure 4A). Both FCU PCR and IgM blood antibody also showed superior PPV (100%, Figures 4.B,C), indicating that for positive results indicated by these test variables, there is a high probability that the results would be truly positive–as indicated by the designated gold standard doctor-collected vaginal swab PCR. However, NPV results for these two test variables were slightly less so; FCU PCR results revealed an NPV of 96.30%, while IgM antibody results had an NPV of 89.66%. This means that given the negative test results, there is a slight probability that they are truly positive (3.7% and 10.34% FOR respectively). These results were somewhat consistent with the sensitivity analyses, where both FCU PCR and IgM antibody had a probability of producing false negative results. On the contrary, though IgG antibody was shown to have high NPV, its PPV stood at 33.33% (Figure 4D), which further corroborated earlier results.

Reiterating recommendations set forth in the “*Recommendations for the Laboratory-Based Detection of Chlamydia trachomatis and Neisseria gonorrhoeae–2014*” by the Centres for Disease Control and Prevention, the use of NAATs remain the recommended laboratory testing methodology for the detection of *C. trachomatis* (Papp et al., 2014). Results from the current study are consistent with this recommendation, but also further highlights the self-collected vaginal swab’s additional advantages over other conventional diagnostic tests (Table 1). Apart from its superior sensitivity and specificity matching the results of doctor-collected vaginal swab samples, it is also superior in terms of storage and transport. FDA-cleared specimen transport and storage conditions for urine specimens for commercially available NAATs commonly rely on refrigeration or the addition of specific buffer solutions as a general rule of thumb (Papp et al., 2014). The currently studied self-collection tool showed promising results even at slightly relaxed transport conditions compared to first catch urine. They are dry stored in a tube and therefore have no risk of spillage, which can be more convenient and user-friendly for women. As for blood antibodies, not only are they invasive, their results were not as accurate as those of the self-collection tools. Both *C. trachomatis* IgM and IgG showed high proportions of false negatives and false positives when compared to the standard CDC recommended NAAT test to be considered for diagnosis aid. It is also important to note that it is possible that blood antibodies are evidence of a previous infection–as is the case with a COVID-19 infections (Kobashi et al., 2021)–whereas vaginal swab and FCU PCR are evidence of a recent or current infection, as NAATs directly detect specific nucleic acid sequences in a sample. Another possible interpretation for cases where PCR was positive, but antibodies were negative, is that the body might not have developed the antibodies for a current infection, as was hypothesised and reported in a past epidemiological study (Land et al., 2010). Moreover, this also suggests that whilst it is possible that *C. trachomatis* serology can be a marker for active infection (Perhar et al., 2020), it might find more utility in cases where patients have developed PID and clinicians are trying to determine the cause post-complication. Regardless, current results have shown that NAATs remain superior compared to blood antibodies in detecting *C. trachomatis* for early treatment purposes.

**TABLE 1:** Quick summary of each of the four diagnostic tests in terms of their cross-tabulation results against doctor-collected vaginal swab PCR.

Diagnostic test	Sensitivity (%)	Specificity (%)	PPV (%)	NPV (%)	Fisher’s exact test (*p*-value)
Self-collected vaginal swab	100	100	100	100	0.000
First catch urine (FCU)	75	100	100	96.30	0.001
Blood IgM antibody	25	100	100	89.66	0.133
Blood IgG antibody	100	69.23	33.33	100	0.018

From a wider perspective, the self-collection tool also has the potential to encourage STD screening amongst young women without compromise to diagnostic accuracy. Looking back at Millstein and others’ study on sources of adolescent anxiety about pelvic examinations, some one of the most common reasons for not getting tested were the invasion of privacy and discomfort (Millstein et al., 1984). The self-collection tool provides an opportunity for clinicians to administer a test that is convenient and has a lowered risk of violating the patients’ privacy by letting them take vaginal swab samples by themselves, as they have been shown to garner more acceptance compared to doctor-collected methods in earlier studies (Wiesenfeld et al., 2001; Doshi et al., 2008). Moreover, results were consistent with findings by studies such as Shaffer and others’, who have demonstrated the ability of self-collected samples in identifying positive cases through NAAT (Shafer et al., 2003). This means that in addition to ease of sample collection, the self-collection tool has been engineered to be at least as good as its doctor-collected counterpart and could therefore also potentially reduce the number of false diagnoses. As self-collection tools have also been shown to improve STD testing compliance among young women (Wiesenfeld et al., 2001), the development of a tool such as this can also hopefully reduce the number of silent transmissions, especially among asymptomatic cases. The study can benefit from further analyses such as those done by Chen et al. (2007) and others and Wiesenfeld and others (Wiesenfeld et al., 2001), who looked at the predictors of testing positive for STD through multivariable logistic regression analysis. Elements of subjects’ attitudes, affective responses, patient history, STD risk factors, etc. Administered *via* self-administered questionnaires (Millstein et al., 1984; Wiesenfeld et al., 2001) can also be incorporated to look at their relationship with results obtained by the self-collected vaginal swab device. As the current pilot study is limited in its focus on initial clinical validation, and has limited sample size, a more in-depth analysis such as this would have to be saved for a future work. However, there is still evidence in the current study to suggest that the device can potentially be used as a convenient self-collected vaginal swab device to aid clinicians in *C. trachomatis* diagnosis.

## Conclusion

There is evidence to suggest that the currently studied vaginal swab self-collection tool is at least as good as the designated standard for *chlamydia* diagnosis. More than that, its convenience and its ease of use, storage, and transport potentially makes it a more powerful tool in the clinical diagnosis of *C. trachomatis*. All of these could therefore potentially help in the proper treatment of this curable sexually transmitted disease by encouraging young adolescents to get screened without worry of potential discomfort during examination. Future studies can look into a large-scale clinical validation study involving a larger pool of participants and include other preventable endemic bacterial STDs such as *gonorrhoea* and *trichomoniasis* to study the limits of the current device in the fight against preventable sexually transmitted diseases.

## Data Availability

The raw data supporting the conclusions of this article will be made available by the authors, without undue reservation.
